# Systemic injection of an H4 receptor agonist induces a decrease in CREB and pCREB levels in the cerebellar vermis and prefrontal cortex in mice

**DOI:** 10.1590/1414-431X20198334

**Published:** 2019-04-25

**Authors:** C.E.M. Fernandes, K.R. Serafim, A.C.L. Gianlorenço, R. Mattioli

**Affiliations:** Laboratório de Neurociência, Departamento de Fisioterapia, Centro de Biologia, Ciências e Saúde, Universidade Federal de São Carlos, São Carlos, SP, Brasil

**Keywords:** CREB, pCREB, VUF-8430, Western blotting, Emotional memory, Mice

## Abstract

Studies have shown that an injection with the histamine H4 receptor agonist VUF-8430 modulates emotional memory processes. In the present study, the aim was to verify if intraperitoneal (*ip*) injection of VUF-8430 (500 ng/kg) in mice affects the synthesis of proteins required for memory consolidation processes by activating the phosphorylation of CREB (pCREB) in classical structures linked to emotional memory (prefrontal cortex, amygdala, and hippocampus) and the cerebellar vermis, a structure that has also been recently implicated in emotional memory. The results obtained using western blot analysis demonstrated that VUF-8430 induced a decrease in CREB and pCREB levels in the cerebellar vermis and prefrontal cortex, suggesting that this dose impaired the activation of cell signaling pathways in these structures. There was no change in protein expression in the amygdala and hippocampus. Our results are preliminary, and further investigations are needed to investigate the role of the H4 receptors in the central nervous system.

## Introduction

The histaminergic system has been shown to modulate emotional memory through different receptors, including at least four distinct subtypes of G-protein coupled receptors (H1–H4) that mediate the biological effects of histamine (HA) (1). These four receptor subtypes differ in their preferred G-proteins for binding and thus, upon ligation with HA, activate different signal transduction pathways (2). HA excites postsynaptic neurons through the activation of H1 and H2 receptors, modulating cognition, emotional processes, and the circadian rhythm (3). The H3 receptor exerts inhibitory presynaptic activity on the synthesis and release of HA and is related to changes in behavior and locomotion (4). The H4 receptors were previously associated with the peripheral nervous system, and it was only recently discovered that these receptors are also expressed in the central nervous system (CNS) (1).

VUF-8430 demonstrated high affinity for the H4 receptors as an agonist, although it has an affinity for the H3 receptors and can serve as a pharmacological tool to validate the H4 receptor as a new drug target in the CNS (5). A study conducted by Galeotti et al. (6) demonstrated that intracerebroventricular (*icv*) microinjections of this H4 receptor agonist reversed emotional memory deficits induced by scopolamine in mice subjected to an inhibitory avoidance task (IAT), which is a model related to fear expression. A recent study conducted by Fernandes et al. (7) demonstrated that microinjections of VUF-8430 in the cerebellar vermis of mice impaired memory consolidation in two models of learning and emotional memory: the elevated plus maze (EPM), which is a model for assessing anxiety, and the IAT. A study by Sanna et al. (8) in H4 receptor-knockout mice showed that these receptors modulate various neurophysiological functions, such as anxiety, locomotor activity, and nociception, indicating the importance of the integrity and functionality of the H4 receptors in the histaminergic regulation of neuronal functions.

As is well-known, the phosphorylation-induced activation of the cellular transcription factor cyclic AMP response element binding (CREB) protein is a necessary component of memory formation and cognitive processes (9). In addition to the memory formation, CREB also demonstrated an important role in many complex behaviors, including circadian rhythm, and its activity depends on the encephalic region that is involved (10).

Considering the results obtained by Fernandes et al. (7) that VUF-8430 microinjection into the cerebellar vermis induced an inhibitory effect on memory consolidation in the EPM and IAT models, this study aimed to investigate whether VUF-8430 induces activation of CREB and phosphorylated CREB (pCREB) in the following structures: cerebellar vermis, prefrontal cortex (PFC), amygdala, and hippocampus.

## Material and Methods

### Animals

The experimental subjects were adult male Swiss mice (Federal University of Sao Carlos, Brazil) aged 5–6 weeks and weighing 25–30 g. The mice were housed in groups of 5 per cage (28×18×11 cm) and were maintained under a 12-h light/dark cycle (lights on at 7 a.m.) in a controlled environment at a temperature of 23±1°C and a relative humidity of 50±5%. Food and water were provided *ad libitum*. All mice were naive at the beginning of the study.

All procedures were approved by the Ethics Committee on Animal Experimentation of the Federal University of Sao Carlos (Process #8336250515) and complied with the US National Institutes of Health Guide for the Care and Use of Laboratory Animals.

### Drug

VUF-8430 (Sigma Chemical Co., USA), an H4 receptor agonist, was dissolved in sterile 0.9% saline solution and was administered via intraperitoneal (*ip*) injection at a dose of 500 ng/kg, adapted from previous studies conducted in our laboratory (7) and by Lim et al. (5).

### Experimental procedure

Animals received *ip* injections of 0.9% saline (SAL group) or 500 ng/kg VUF-8430 (VUF group). After 2 h, their brains were removed, and the samples were stored for western blotting. For this analysis, 5 animals per group were used for each brain structure of interest: cerebellar vermis, prefrontal cortex, amygdala, and hippocampus.

After brains were extracted, they were frozen in isopropane (–50°C) and stored until dissection and processing with a cryostat at a temperature of –20°C, following coordinates from the mouse brain atlas of Franklin and Paxinos (11). Nuclear protein extracts from the cerebellar vermis, prefrontal cortex, amygdala, and hippocampus were solubilized in a sample buffer (0.125 M Tris base; 4% SDS; 20% v/v glycerol; 0.2 M DTT; 0.02% bromophenol blue, pH 6.8) at a concentration of 10 μg/15 μL per well and boiled for 5 min at 95°C. Protein fractions were electrophoresed for 80 min at 130 V on 12% SDS-polyacrylamide gels (acrylamide/bisacrylamide 29:1, 10% SDS). At the end of the separation, the proteins present on the gel were transferred to a nitrocellulose membrane immersed in a transfer buffer (25 mM Tris base; 192 mM glycine; 10% methanol) (1 h at 110 V). The membranes were stained with Coomassie blue solution (1% Coomassie blue, 10% acetic acid, 50% methanol) for 1 min and washed in bleach solution (5% acetic acid, 40% methanol) for 2 min with stirring (for verification of the efficiency of the transfer procedure). After removal of the dye, the membranes were incubated for 1 h at room temperature in TTBS blocking solution (10 mM Tris base; 150 mM NaCl) with 5% skim milk and 0.05% Tween 20 to block nonspecific antibody binding. After this step, the membranes were washed in a TTBS solution (TBS, 0.05% Tween 20) and incubated with anti-CREB-1 (1:1000, code sc-377154; Santa Cruz Biotechnology, Inc., USA) or anti-CREB primary antibodies (1:750, code MAB5432; Merck/Millipore, Germany) diluted in TTBS with 3% skim milk overnight at 4°C. The membranes were washed with TTBS and incubated with a peroxidase-conjugated goat anti-rabbit secondary antibody (code 111-035-144; Jackson ImmunoResearch Laboratories, Inc., USA) for 1 h at room temperature with constant stirring. Membranes were developed using the ECL Chemiluminescence kit (Thermo Fisher Scientific, USA). For this purpose, membranes were bathed in ECL for 1 min and exposed to ECL-sensitive film (Thermo Fisher Scientific) for an appropriate length of time for each antibody.

α-tubulin levels were detected to determine whether there was variation in the amount of protein applied to each gel. The membranes were initially subjected to a bath of 5% acetic acid solution for 10 min followed by washing for 3X/10 min in TTBS with constant stirring. Then, the membranes were blocked in TTBS with 5% skim milk and subsequently incubated with an α-tubulin monoclonal mouse antibody (1:10000, code T6199; Sigma-Aldrich Corp.) followed by incubation with a peroxidase-conjugated anti-mouse secondary antibody produced in sheep (1:600, code NA931; GE Healthcare Life Sciences, England).

Autoradiographs were scanned in transparency mode and the intensity of the bands was quantified with the Image-Master program (Amersham Pharmacia Biotech). To analyze CREB and pCREB levels, the data obtained from each band were normalized by the respective α-tubulin value. The pCREB/CREB ratio was determined, and these values are reported as a percentage increase compared to the control group (equivalent to 100% binding).

### Statistical analysis

All data were tested for normality, variability, and homogeneity of variance (using Levene's tests). Student's *t*-tests were performed for analysis between the two independent groups: control (SAL) and treatment (VUF). In all cases, a P value<0.05 was considered significant.

## Results

**Figure 1.**
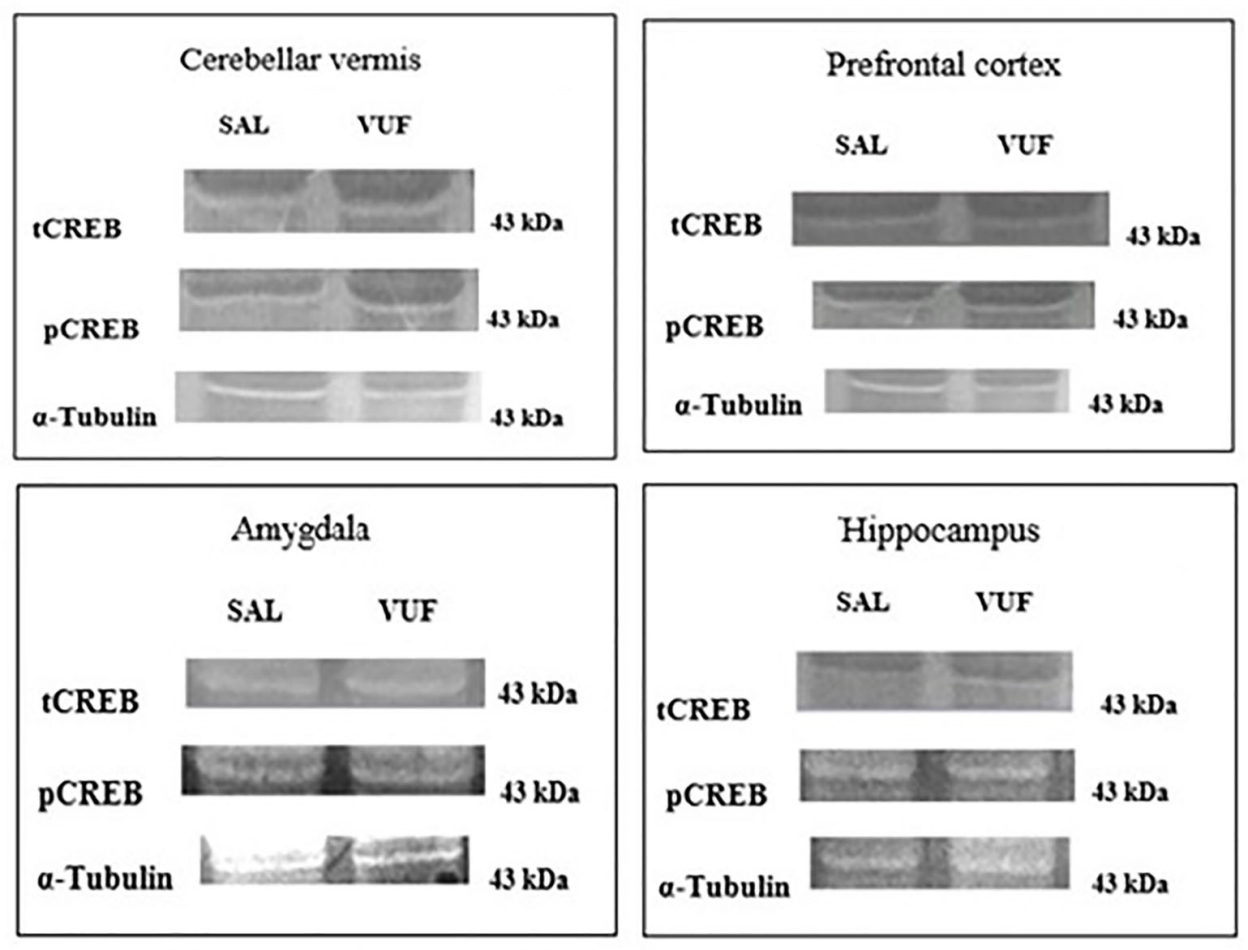
Representative membranes with fluorescence bands from the western blot analysis of total CREB (tCREB), phosphorylated CREB (pCREB), and α-tubulin in the cerebellar vermis, prefrontal cortex, amygdala, and hippocampus in mice. SAL: 9% saline group; VUF: 500 ng/kg VUF-8430-treated group.

Figure 1 shows representative fluorescent bands obtained after immunoblotting using a western blot assay for total CREB (tCREB), pCREB, and α-tubulin in the brain structures. The band was labeled by the anti-CREB monoclonal antibody (molecular weight: 43 kDa).

**Figure 2.**
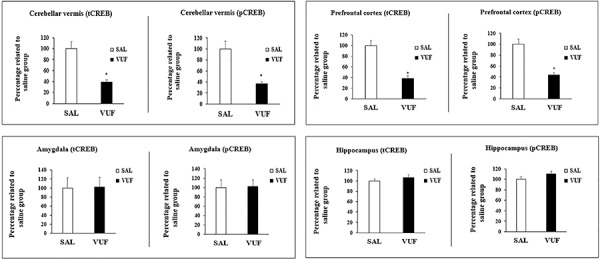
Percentages of total CREB (tCREB) and phosphorylated CREB (pCREB) of the 500 ng/kg VUF-8430-treated group (VUF) compared to the 9% saline control group (SAL) in the following structures: cerebellar vermis, prefrontal cortex, amygdala, and hippocampus. Data in the histograms are reported as the means±SE (n=5 mice/group). *P<0.05, Student’s *t*-test.

Figure 2 shows the results of western blot analysis of tCREB and pCREB levels in the brain structures of a sample collected after *ip* injection of SAL or VUF (500 ng/kg) in mice. Significant differences were found in the tCREB value for the following structures: cerebellar vermis (t(8)=2.95, P<0.018) and prefrontal cortex (t(8)=3.07, P<0.015). No significant difference was found between the SAL and VUF groups in the amygdala (t(8)=−0.44, P>0.671) or the hippocampus (t(8)=−0.45, P>0.659). With regards to pCREB levels, significant differences were found in the cerebellar vermis (t(8)=3.21, P<0.012) and the prefrontal cortex (t(8)=2.85, P<0.021), but not in the amygdala (t(8)=−0.69, P>0.504) or hippocampus (t(8)=−0.49, P>0.631).

## Discussion

The results obtained in the present study demonstrated that *ip* administration of VUF-8430 induces a decrease in CREB and pCREB expression in the cerebellar vermis and prefrontal cortex of mice, suggesting that this dose impaired the activation of cell signaling pathways in these structures. There was no change in the expression of these transcription factors in the amygdala and hippocampus.

Sanna et al. (8) suggested that the H4 receptor subtype did not play a prominent role in the histaminergic modulation of memory processes; however, a recent study performed in our laboratory (7) showed that intravermis microinjections of VUF-8430 induced a deficit in memory consolidation in mice subjected to the EPM and IAT tests. On the other hand, a study by Galeotti et al. (6) showed that VUF-8430 microinjected *icv* in mice reversed deficits in emotional memory induced by scopolamine in the IAT, but the microinjection alone did not show effects on emotional memory. In the present study, we showed that the acute administration of VUF-8430 induced a significant repression of CREB signaling expression in the cerebellar vermis that is associated with cognitive impairment, which could be related to the biochemical events associated with cognitive impairment found in our previous study (7).

In addition to the role of CREB in the formation of memories (12), it is known that CREB phosphorylation is involved in many adaptive behaviors that we cannot exclude triggering the transcription of circadian genes that will act on the circadian rhythm and could influence an animal's performance (10). In the present study, we injected saline or drug solutions in animals at the same time of day to minimize this bias.

In our study, there was also a decrease in CREB and pCREB expression in the prefrontal cortex. The attenuation of the signaling pathway in the cerebellar vermis induced by VUF-8430 could have decreased the stimulation of the prefrontal cortex, since the output of the cerebellum targets not only cortical motors areas but also regions related to emotional modulation, such as the prefrontal cortex, which is involved in attention and cognitive control (13). According to Chen et al. (14), genetic and pharmacological attenuation of CREB expression impairs the excitability of neural networks and cognitive functions.

Albeit the decrease in CREB and pCREB levels in the cerebellar vermis and the PFC, our results also demonstrated that the histaminergic H4 receptor agonist had no significant impact regarding induction of CREB phosphorylation in the hippocampus and the amygdala. The amygdala and the hippocampus are anatomically connected, and they interact functionally during emotional memory coding (15). The amygdala basolateral nucleus modulates the function of the hippocampus during fear conditioning and promotes a dynamic interaction between these structures, which mutually influences neuroplasticity and promotes the modulation of learning and memory processes (16). In our study, the decreased expression of CREB in the cerebellar vermis could have impaired the activation of signaling pathways in the amygdala and hippocampus. Furthermore, the lack of effect of VUF-8430 in the hippocampus and amygdala could be related to the dose used in the present study.

The individual effect that HA induces on a given cell type is determined by the combination of expressed histaminergic receptors subtypes, thus H1 and H4 receptors mediate the activation of CREB in a cooperative manner (17). The mechanism underlying the combined activation of these receptors that results in synergistic CREB phosphorylation still has to be elucidated and further investigations are required to determine the interaction points of both signaling pathways on a molecular level (18).

The H4 receptors upon coexpression with the H1 receptors probably lose their ability to induce intracellular calcium mobilization based on an interaction of the signaling pathways emerging at the individual receptors (18). According to Beermann (18), the H4 receptor is a G0/i coupled receptor; therefore, its activation reduces the cAMP signaling pathway and CREB/pCREB expression by this pathway. In fact, it would not be expected that the main outcome is an increase in CREB expression by these receptors. In the present study, we observed a decrease in CREB and pCREB expression in the cerebellar vermis and prefrontal cortex induced by an H4 receptor agonist in mice.

In addition to the high affinity demonstrated by the drug to the H4 receptors, VUF-8430 also has an affinity to the H3 receptors as an agonist (5). We believe that the effect was, at least in part, due to the H4 receptors based on the comparison of different results obtained from previous studies involving the H3 receptors (19,20) and our previous study (7). However, there is a need for further studies to investigate the role of the H4 histaminergic receptors in the CNS, since this study was the first to investigate the action of this agonist via western blot analysis of protein expression in structures linked to memory. Furthermore, the pharmacological activation of specific cellular signaling pathways may be used to evaluate the therapeutic potential of drugs for the treatment of neurological diseases and could enhance knowledge regarding the neurobiological basis of memory formation.
